# Neurocognitive and Neuroplastic Mechanisms of Novel Clinical Signs in CRPS

**DOI:** 10.3389/fnhum.2016.00016

**Published:** 2016-01-27

**Authors:** Anoop Kuttikat, Valdas Noreika, Nicholas Shenker, Srivas Chennu, Tristan Bekinschtein, Christopher Andrew Brown

**Affiliations:** ^1^Department of Rheumatology, Addenbrooke’s Hospital, Cambridge, UK; ^2^Cognition and Brain Sciences Unit, Medical Research Council, Cambridge, UK; ^3^Department of Clinical Neurosciences, University of Cambridge, Cambridge, UK; ^4^Department of Psychology, University of Cambridge, Cambridge, UK; ^5^CamPAIN Group, Department of Anaesthesia, University of Cambridge, Cambridge, UK

**Keywords:** perceptual disturbance, neuroimaging, cognition, plasticity, neuroinflammation

## Abstract

Complex regional pain syndrome (CRPS) is a chronic, debilitating pain condition that usually arises after trauma to a limb, but its precise etiology remains elusive. Novel clinical signs based on body perceptual disturbances have been reported, but their pathophysiological mechanisms remain poorly understood. Investigators have used functional neuroimaging techniques (including MEG, EEG, fMRI, and PET) to study changes mainly within the somatosensory and motor cortices. Here, we provide a focused review of the neuroimaging research findings that have generated insights into the potential neurocognitive and neuroplastic mechanisms underlying perceptual disturbances in CRPS. Neuroimaging findings, particularly with regard to somatosensory processing, have been promising but limited by a number of technique-specific factors (such as the complexity of neuroimaging investigations, poor spatial resolution of EEG/MEG, and use of modeling procedures that do not draw causal inferences) and more general factors including small samples sizes and poorly characterized patients. These factors have led to an underappreciation of the potential heterogeneity of pathophysiology that may underlie variable clinical presentation in CRPS. Also, until now, neurological deficits have been predominantly investigated separately from perceptual and cognitive disturbances. Here, we highlight the need to identify neurocognitive phenotypes of patients with CRPS that are underpinned by causal explanations for perceptual disturbances. We suggest that a combination of larger cohorts, patient phenotyping, the use of both high temporal, and spatial resolution neuroimaging methods, and the identification of simplified biomarkers is likely to be the most fruitful approach to identifying neurocognitive phenotypes in CRPS. Based on our review, we explain how such phenotypes could be characterized in terms of hierarchical models of perception and corresponding disturbances in recurrent processing involving the somatosensory, salience and executive brain networks. We also draw attention to complementary neurological factors that may explain some CRPS symptoms, including the possibility of central neuroinflammation and neuronal atrophy, and how these phenomena may overlap but be partially separable from neurocognitive deficits.

## Introduction

Complex Regional Pain Syndrome (CRPS) is a chronic, debilitating pain condition that usually arises after trauma to a limb. It is characterized by disproportionate pain and variable combinations of sensory (allodynia, hyperalgesia), vasomotor (temperature changes or asymmetry, skin color changes or asymmetry), sudomotor (sweating changes or asymmetry), edema, trophic (thin glossy skin, abnormal hair growth, coarse nails), and motor (weakness, decreased range of motion, tremor, dystonia) changes. In up to 10% of cases, there is no obvious trauma reported (Turner-Stokes and Goebel, 2011). Although CRPS is primarily a limb-confined condition, it has also been reported in other body parts including face (Melis et al., 2002). The precise etiology of this enigmatic condition remains unexplained. Based on observational evidence of aberrant inflammation, vasomotor dysfunction, and cerebral cortical changes, it has been proposed that these factors account for the main features of CRPS but may occur to a different extent depending on individual susceptibility (Marinus et al., 2011), thus accounting for the clinical heterogeneity of the condition.

A number of perceptual disturbances reported in patients with CRPS may serve as both novel clinical signs of the condition and markers for underlying biological mechanisms that can be targeted for treatment. Prominent examples include finger misperception, impaired hand laterality recognition, astereognosis, and abnormal body scheme (Förderreuther et al., 2004; Moseley, 2004; Cohen et al., 2013). CRPS patients may also report unusual symptoms such as “feeling of foreignness” and wish to amputate the affected limb (autotomy wish) (Galer and Jensen, 1999). Overall, the evidence points to patients with CRPS having difficulty with the mental representation of their affected limb. However, as outlined in this review, despite many neuroimaging studies of the sensory and motor systems in CRPS, the pathophysiological mechanisms underlying perceptual disturbances remain unknown. We outline a number of approaches to investigating the origin and role of somatosensory perceptual disturbances in CRPS, with a particular focus on the role of top-down (expectancy-related) mechanisms in perception, a relatively unexplored topic in the CRPS literature. We make recommendations for the investigation of the role of top-down mechanisms in CRPS and suggest that this approach may be useful in the delineation of neurocognitive phenotypes that improve our understanding of the heterogeneity of the condition and its causal mechanisms.

## Disturbances of the Body Scheme in CRPS

“Body scheme” is a term used to define the dynamic, real-time, representation of one’s own body. This representation is generated by proprioceptive, somatosensory, vestibular, and other sensory inputs and is integrated with motor systems for control of action in a way that is normally automatic and seamless. Neurological studies indicate that disturbance of body scheme may be caused by abnormal functioning of various parts of cerebral cortex, including the somatosensory (Hari et al., 1998), parietal (Daprati et al., 2010), insular (Karnath and Baier, 2010), and frontal cortices (Weijers et al., 2013). It thus seems that body scheme can be disturbed at different stages or levels of neuronal integration, from the processing of the early sensory inputs to the body scheme integration and spatial orientation in the parietal cortex to the reportable conscious awareness of own body supported by the executive frontal functions. Recent electroencephalography (EEG) studies suggest that each of the early sensory, the mid-latency, as well as the late cognitive stages of neuronal processing contribute to somatosensory awareness (Auksztulewicz and Blankenburg, 2013; Adhikari et al., 2014), which is one of the prerequisites for an intact body scheme. The presence of a frontal component to the pathophysiology seen in patients with CRPS is indirectly supported by the effectiveness of the treatment of subjective CRPS symptoms by cognitive behavioral therapy (De Jong et al., 2005) and acceptance-based approaches (Cho et al., 2013), which function through influences on frontal cortices (Etkin et al., 2005; Brown and Jones, 2013). The possibility of a mechanistic role for aberrant frontal cortical processes in CRPS signs and symptoms is a topic we explore in depth in the latter sections of this review.

One aspect of body scheme is representation of the position of the limb in peri-personal space. Many CRPS patients lack awareness of the position of their limb in space (Lewis et al., 2007) and have difficulty recognizing the laterality of a pictured image of a hand as either left or right (Parsons, 1987). There is also evidence for delayed hand laterality recognition on the affected side that in one study was related to symptom duration and to the pain that would be evoked by executing the movement (Moseley, 2004), pointing to deficits in the ability to represent the position of the limb in space. Interestingly, a single-case study (Bultitude and Rafal, 2010) revealed that unawareness of limb position can occur before the onset of pain symptoms in CRPS, suggesting that perceptual disturbances may be a marker for the pathophysiological mechanisms preceding chronic, limb pain, rather than being a consequence of pain.

Other perceptual assessments have focused on the ability to recognize the somatic location and identity of objects touching the skin of the affected limb, revealing deficits in these finer-grained perceptual judgments in CRPS. Förderreuther et al. (2004) found that 48% of patients with CRPS had an impaired ability to identify the fingers of the affected hand. In contrast, the ability to identify fingers on the unaffected hand was impaired in only 6.5% of patients. Impaired identification of the fingers was not related to the affected side of the body (left vs. right) of CRPS. The study authors also reported that all patients stressed that their difficulties naming the fingers could not be explained by reduced perception of the cotton swab. This provides preliminary evidence for no deficit in afferent transmission of cutaneous sensation, but more likely a change in the way the brain constructs a spatial representation of the limb, similar to the concept of a deficit in the body scheme.

A seemingly related phenomenon, astereognosis, is defined as the inability to identify an object by touch (without visual input) despite having intact cutaneous sensation. Classically, this is reported in patients who have had stroke mainly affecting the parietal lobe, but this has also been reported in some patients with CRPS, including 64% of patients in one study (Cohen et al., 2013). In addition, neurocognitive dysfunctions thought to be similar to the neurological neglect caused by post-stroke damage to the right parietal lobe have been reported in CRPS, and the term “neglect-like” has been used to describe them (Galer et al., 1995). For example, some CRPS patients perceive their own affected limb to be “foreign” and not belonging to them and this is referred to as “cognitive neglect.” Similarly, some CRPS patients need to focus mental and visual attention in order to move their affected limb (“motor neglect”).

Together, these findings suggest possible parietal and frontal lobe involvement in the perceptual disturbances of CRPS and specifically deficits in the ability to represent the location, orientation, and structure of the affected limb. However, it is presently unclear the extent to which these different manifestations of neurocognitive dysfunction in CRPS tend to co-occur within individual patients with CRPS, whether they share overlapping mechanisms, or what pathophysiological mechanisms underpin them, e.g., disturbances in the early (parietal) stages of processing or abnormalities in the latter (frontal) stages of bodily awareness. Neuroimaging research, which we discuss in the next sections, has to date been conducted largely independently of observations of the various perceptual disturbances described above. Hence, the pathophysiological mechanisms of these salient perceptual disturbances remain unknown.

## Neuroimaging of Somatosensory Representations in CRPS

Among the more compelling evidence of aberrant neurophysiology in CRPS is that of somatosensory and motor cortical plasticity, which is often assumed to be the underlying biological cause of body perceptual disturbances in CRPS. Studies of cortical changes in regions representing somatic sensation, namely the primary (S1) and secondary (S2) somatosensory cortices, were inspired by earlier studies of the effects of alterations of afferent input (as occur in many types of chronic pain) on cortical reorganization of sensory maps. In monkeys, digit amputation resulted in shrunken representation in area 3b of SI cortex of the corresponding finger (Merzenich et al., 1984), while subsequent human work using magnetoencephalography (MEG) found that upper limb amputation also caused the face area of S1 cortex to expand into the former hand area (Flor et al., 1995). Critically, these latter findings predicted the intensity of concurrent phantom limb pain, consistent with previous work finding similar correlations between back pain and S1 cortical reorganization (Flor et al., 1997) [however, also see Makin et al. (2013, 2015) who present contradictory evidence of a lack of invasion of the former hand area by the lip area and a lack of correlation of cortical reorganization with pain intensity]. Together, these results implicated similar somatosensory cortical changes in patients with other types of chronic pain, including neuropathic pain and CRPS.

Subsequently, a number of studies (reviewed in the next section) used MEG or EEG with source imaging, or functional Magnetic Resonance Imaging (fMRI), to investigate somatosensory cortex representations in CRPS. EEG and MEG data are most commonly interrogated to identify changes in electrical or field potentials generated by the coordinated activity of assemblies of cortical pyramidal cells. The majority of studies adopted similar EEG/MEG methods to that of the aforementioned work in phantom limb and chronic low back pain. Indeed, EEG/MEG methods are naturally powerful techniques to resolve, with high temporal resolution, early somatosensory responses that are most closely related to afferent inputs.

Studies in patients with CRPS have mostly assessed averaged *time-locked* signals (evoked potentials or fields), which are the stimulus-driven cortical changes that have consistent onset latency in relation to the stimulus across many experimental trials. These averaged stimulus-evoked responses can be quantified or further processed in a number of ways, and studies of CRPS have to date focused on one or a number of outcomes: (1) the amplitude of the evoked signal, representing the sum of the activity of (predominantly excitatory) cortical neurons, to investigate possible deficits in afferent processing of somatosensation, (2) the evoked signal’s latency/timing with respect to the stimulus, to investigate possible delays to the afferent signals reaching the brain, (3) habituation/suppression of the amplitude of the evoked signal by multiple stimulus repetitions, to investigate the possibility of deficits in intracortical inhibition, and (4) source modeling of the evoked responses to reveal the spatial location of cortical generators of the signal, to investigate possible changes in the location of cortical representations of the affected limb. In addition to these methods, *non-time-locked* neuronal oscillations in different frequencies can be measured. fMRI studies have also been conducted in which the magnitude of the evoked signal and cortical spatial representations have been resolved.

## Somatosensory Spatial Representations

Of the lines of neurological investigation conducted in CRPS as summarized above, a recent meta-analysis by Di Pietro et al. (2013) confirmed that the strongest evidence of aberrant neurological changes in CRPS is plasticity in cortical representations of the affected limb, manifesting as a reversible shrinkage of the somatosensory cortex. In the meta-analysis, pooled data from four MEG studies (Juottonen et al., 2002; Maihöfner et al., 2003; Sinis et al., 2007; Vartiainen et al., 2008) and one EEG study (Pleger et al., 2004) were examined. Meta-analysis is highly desirable with respect to the above studies given the small sample sizes, ranging from single-subject analyses (Sinis et al., 2007) to more commonly between 6 and 12 patients. The authors of the meta-analysis reported that the evidence supported the hypothesis that S1 representations of the body were reduced on the affected hand compared to the unaffected hand in CRPS.

A shrinkage in the Penfield’s homunculus (Penfield and Boldrey, 1937) would provide a compelling explanation for many of the perceptual disturbances seen in CRPS. Cortical reorganization may disrupt the internal body map and impair performance on the tasks requiring the identification of somatosensory information and coding of body posture. However, the evidence supporting this hypothesis has limitations that should be acknowledged, which we discuss in detail below. First, there are important methodological caveats of EEG/MEG for assessing cortical reorganization. Second, the studies included in the meta-analysis suffered from a high risk of bias, which more recent work [not included in the meta-analysis by Di Pietro et al. (2013)] has addressed, finding conflicting results.

Regarding methodological limitations of EEG/MEG, an important issue is that the reported spatial changes in somatosensory responses in comparing thumb and little finger digits (typically in the region of 5 mm on average) are comparable to or smaller than the estimated spatial resolution and accuracy of the best available source modeling methods with MEG and EEG based on *simulated* data (Darvas et al., 2005; Yao and Dewald, 2005), which must therefore be considered optimistic when applied to clinical data. With clinical data, the accuracy of the source model may be affected by unknown/unmodelled concurrent neural responses such as those involved with top-down modulation from higher-order cortical regions. Subject motion during recording/scanning, which is more likely in patients with more severe symptoms, can reduce data quality and introduce artifactual effects that may underestimate the observational parameters. The introduction of “noise” from the above sources risks biasing results, especially in studies with small samples sizes.

Corroboration of representation changes measured with MEG/EEG by complementary techniques, such as fMRI, is essential for the evidence to conclusively converge. However, the results of the relatively few currently published fMRI studies investigating somatosensory cortical plasticity in CRPS are equivocal about the precise cortical changes taking place. Pleger et al. (2005) found support for the EEG/MEG findings already discussed. However, as detailed by Di Pietro and colleagues in their meta-analysis (Di Pietro et al., 2013), the majority of EEG, MEG, and fMRI studies in CRPS to date have a potential for bias arising from the selective reporting, unclear outcomes and unblinded assessments. In order to address this, data from a more recent fMRI study (Di Pietro et al., 2015) was analyzed blind to the group (CRPS patients or healthy controls) and hand (affected or unaffected). Contrary to previous findings, CRPS was associated with an enlarged representation of the healthy hand, not a smaller representation of the affected hand. Consistent recent findings from fMRI studies of cortical reorganization in patients with phantom limb pain also shed doubt on the hypothesis that maladaptive plasticity is the cause of phantom limb pain: patients with greater pain intensity had a more greatly preserved representation of the former hand area, with pain thought to arise from nociceptive or top-down inputs rather than maladaptive plasticity (Makin et al., 2013). Further studies are needed to replicate and confirm these fMRI results in patients with CRPS. As well as minimizing the potential for bias, studies could use multiple converging neuroimaging methods, e.g., EEG combined with fMRI to improve spatial localization of early somatosensory responses.

## The Challenge of Heterogeneity

Another common shortcoming of neuroimaging studies to date is the inclusion of only small numbers of poorly characterized patients. Heterogeneity in terms of clinical presentation is well documented (Marinus et al., 2011). It also appears that there is significant heterogeneity within the CRPS population on the basis of studies of perceptual disturbance in CRPS. For example, finger misperception occurred in 48% of CRPS patients in one study (Förderreuther et al., 2004), and it is unknown to what extent finger misperception overlaps with other deficits. It is possible that there are common pathophysiological mechanisms, cutting across the different types of body perceptual disturbance, which manifest differently on an individual patient basis depending on other biological and psychological susceptibility factors. Alternatively, the mechanisms underlying two different manifestations, for example identifying fingers and recognizing the laterality of a presented hand, may be entirely or largely discrete.

To illustrate, in comparing the phenomena of astereognosis with finger misperception, the functional difference in terms of somatosensory processing can be summarized in terms of a “what” (i.e., objective identification) vs. “where” (discrimination of tactile stimulus location) distinction. Investigations of somatosensory processing with fMRI (Reed et al., 2005) have compared “what” vs. “where” processes, showing differential activation patterns. Tactile object recognition activated frontal as well as bilateral inferior parietal areas. In contrast, tactile object location activated bilateral superior parietal areas. A common gray matter deficit across CRPS patients presenting with different perceptual abnormalities therefore seems unlikely.

On the other hand, investigations of white matter may be warranted. The possibility of a common white matter deficit explaining a constellation of neurocognitive dysfunctions is illustrated by findings from research conducted in patients with Gerstmann syndrome (Gerstmann, 1940). In this syndrome, parietal lobe lesions lead to a tetrad of finger agnosia (difficulty in the naming of fingers), agraphia (difficulty in writing), acalculia (difficulty in performing calculations), and left to right confusion. Neuropsychological studies during open brain surgery found a relation between the Gerstmann tetrad and left parietal cortex and demonstrated a certain degree of proximity and overlap of those cortical sites where electrical stimulation can elicit these symptoms (Morris et al., 1984). More recently, Rusconi et al. (2009) used fMRI and diffusion tensor imaging in healthy subjects to identify that the parietal activation patterns across all four domains consistently connected to a small region of subcortical parietal white matter. Hence, Gerstmann syndrome might arise from disconnection, via a lesion, to separate but co-localized fiber tracts in the subcortical parietal white matter.

**Figure 1:**
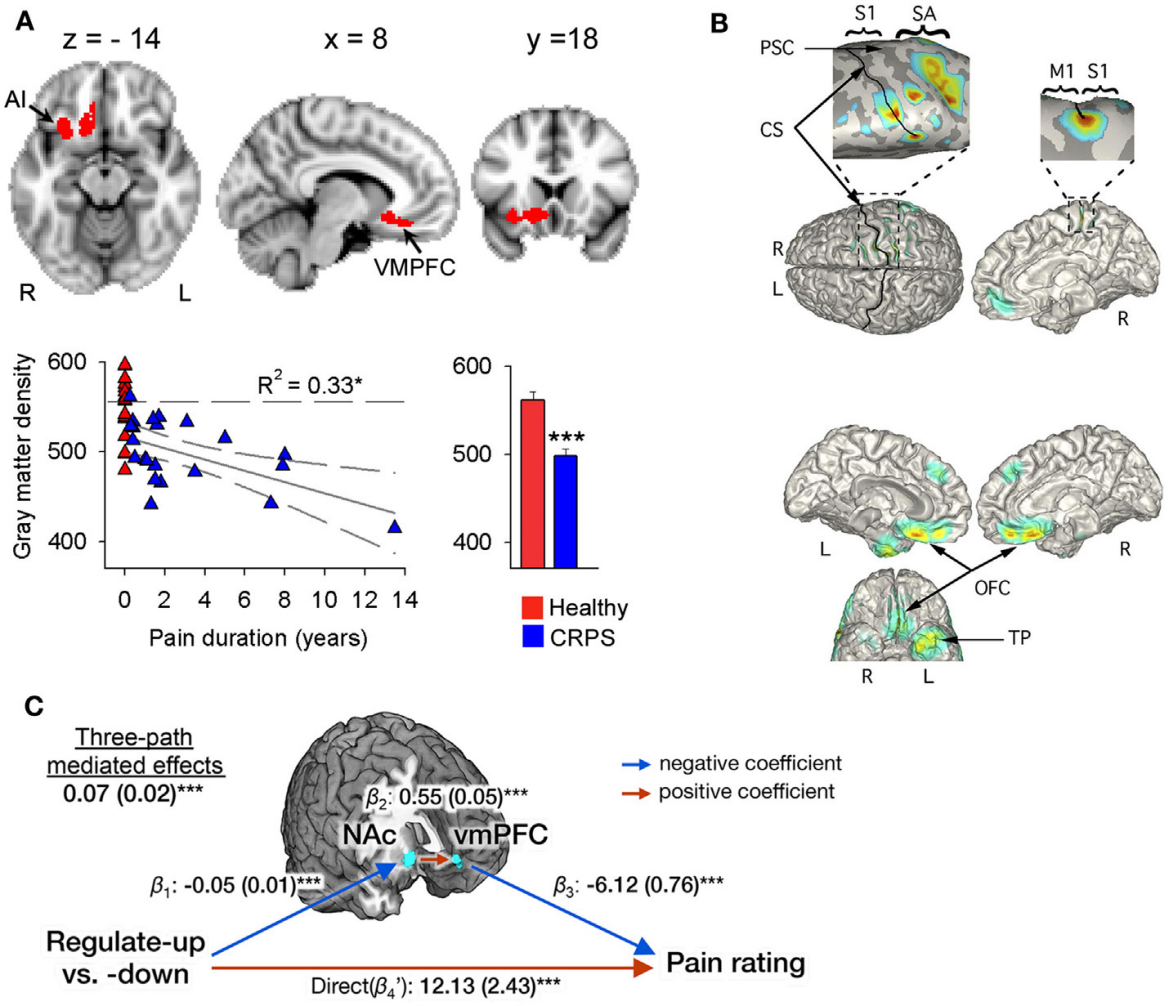
**Evidence for a role of the ventromedial PFC and nucleus accumbens in CRPS and in the self-regulation of pain**. **(A)** Brain regional gray matter density, as measured with voxel-based morphometry (VBM), is decreased in patients with CRPS relative to healthy controls in the right hemisphere (red), spanning the ventromedial PFC, anterior insula (AI), and nucleus accumbens (arrows). The scatter plot shows that this decreased gray matter density is negatively correlated to the number of years the patients have been living with CRPS. Individual healthy control subjects are shown at pain duration = 0. The histogram depicts mean (±SEMs) gray matter density within the cluster in both groups. Reproduced from Geha et al. (2008). **(B)** The localization of MEG-derived independent components (ICs) for a CRPS patient with pain in her left foot and ankle. Top: the localization of the first IC (with frequency spectra in the delta, theta, and beta range) to right S1 and M1 along the central and post-central sulcus, extending to the mesial surface and over the right SA in the superior parietal cortex (see expanded views). Bottom: localization of an IC in the theta range to orbitofrontal cortex bilaterally and left temporal pole. Reproduced from Walton et al. (2010). **(C)** Multilevel three-path mediation analysis with the ventromedial PFC and nucleus accumbens as *a priori* regions-of-interest, showing that these regions formally mediate the effect of instructions to voluntarily upregulate and downregulate pain perception on subjective pain ratings. Reproduced from Woo et al. (2015).

In a similar fashion, it has been suggested that perceptual disturbances in CRPS arise from changes within the parietal lobe (Cohen et al., 2013), where a matrix of a coherent body scheme may arise (Daprati et al., 2010). In support of this hypothesis, an fMRI study of activations relating to cold- or brush-induced allodynia in pediatric CRPS patients identified right parietal lobe involvement (Lebel et al., 2008). However, it is far from clear that there is a consistent constellation of perceptual disturbances in all patients with CRPS, or within a subgroup that has yet to be defined, that would point to a single unifying mechanism. Indeed, the idea that a parietal lobe deficit might be responsible for CRPS was challenged by a study of gray matter atrophy and white matter reorganization (Geha et al., 2008), in which atrophy in patients with CRPS was found in cluster encompassing right ventromedial prefrontal cortex (PFC), anterior insula, and nucleus accumbens (Figure 1A). The study found co-localized decreases in white matter anisotropy and changes in branching and connectivity of white matter tracts linked to these site-specific gray and white matter abnormalities. Deficits in the parietal lobe, however, were not evident in the patient sample studied. Smaller gray matter volume in ventromedial PFC in normal or pathological states has been observed to relate to poorer performance on tasks requiring cognitive control and decision making (Clark et al., 2008b; Boes et al., 2009). Furthermore, it is interesting to note that recent evidence (Figure 1C) points to the ventromedial PFC and nucleus accumbens as being important in the ability to self-regulate pain (Woo et al., 2015) – more on this in later sections of this review. Overall, these findings suggest that while some CRPS symptoms may be associated with parietal deficits, in other patients altered frontal cortex activity is more apparent, variability that remains to be explained.

Heterogeneity with the CRPS population highlights the importance of characterization/phenotyping and subgrouping of patients for research studies. However, the possibility of there being separate phenotypes within the CRPS population is rarely considered in neuroimaging studies. To date, patient heterogeneity has been considered only in terms of overt sensory and motor symptoms, such as hyperalgesia/allodynia (Maihöfner et al., 2005) and dystonia (Van Rijn et al., 2009). A potentially powerful alternative would be to utilize heterogeneity in body perception that may reveal more subtle and detailed information about the processes and mechanisms of the underlying sensory and motor systems. An analysis of sub-groups of CRPS patients according to the degree of different types of perceptual disturbance would improve our understanding of the pathophysiological underpinnings of these phenomena.

Neuroimaging studies to date have been conducted on small numbers of patients, motivating the need for meta-analytic techniques to draw conclusions. Indeed, CRPS is a rare condition, which makes recruitment of large numbers of patients for research studies in single centers an obvious challenge. The need for identifying and comparing subgroups of patients with CRPS, which will require far larger numbers of patients in a single study than has been conducted to date, underlines this challenge further. Researchers are likely to have to look toward conducting multi-center studies and/or amass databases of patients covering large geographical areas in order to have the scale to compare potentially subtle neurophysiological differences among subgroups. Promising steps have been taken in this regard; for example the CRPS UK Clinical Research Network has established a large registry database of patients (300+ in size at the time of writing) to facilitate epidemiology studies and academic and clinical trials (Shenker et al., 2015).

## Neurological Explanations for Cortical Plasticity

It would be helpful to put the hypothesis of shrinkage of Penfield’s homunculus in CRPS into a broader context and to consider possible mechanisms. It has been proposed that “blurring” of somatosensory maps, i.e., increased overlap between representations of adjacent skin surfaces, would increase the total number of neurons representing the affected body part, leading to generation of the misperception of that body part being larger (swelling) (Haggard et al., 2013). This mechanism was originally discussed in relation to the generation of phantom limb pain, in which case the cause of somatosensory “blurring” is proposed to be deafferentation of C-fibers leading to cortical disinhibition, because C-fibers normally provide continuous inhibition to primary somatosensory cortex (Calford and Tweedale, 1991).

There are numerous lines of evidence supporting the idea that reduced afferent input to the somatosensory cortex increases the perceived size of the corresponding body part and can as a result also increase the perceived painfulness of sensations arising from that body part. Local anesthesia of the thumb produces an increase in the perceived size of the thumb (Gandevia and Phegan, 1999), while anesthetic injections at the dentist make the mouth feel swollen (Türker et al., 2005) and anesthesia of the brachial plexus results in the perception of swelling of the entire arm (Paqueron et al., 2004). Paqueron and colleagues identified that changes in perceived limb size had the same time course as a reduction in sensitivity to pin-prick and thermal sensations, implying the phenomenon is related to reduced cortical input from small diameter Aδ and C-fibers (Paqueron et al., 2004).

However, the hypothesis that disinhibition of somatosensory cortices may underlie somatosensory cortical reorganization and perceptual disturbances in CRPS is not currently well supported. A promising research direction has been the assessment of cortical excitability with EEG/MEG and TMS using a variety of paired-pulse methods. However, while the results of TMS have largely supported the hypothesis of disinhibition of the motor cortices bilaterally in CRPS (Schwenkreis et al., 2003; Eisenberg et al., 2005), EEG/MEG investigations of somatosensory disinhibition in small numbers of CRPS patients have provided mixed results (Van Rijn et al., 2009; Lenz et al., 2011). Furthermore, fMRI evidence suggests greater cortical inhibition in response to affected relative to unaffected limb stimulation of allodynia in pediatric patients with CRPS (Lebel et al., 2008). Future studies should be conducted with larger patient numbers that can identify distinct phenotypic subgroups and associated mechanisms, which may account for some of the variability in the study findings thus far.

The hypothesis of cortical disinhibition in CRPS could be further investigated with respect to possible causal factors. In the case of phantom limb pain, reduced afferent input to the somatosensory cortex is a likely contributing factor. However, there is a lack of consistent evidence suggesting differences in afferent input to the somatosensory cortex in CRPS (Di Pietro et al., 2013). Two out of five EEG or MEG studies (Maihöfner et al., 2004; Pleger et al., 2004) found no consistent differences between affected and unaffected limbs of CRPS patients in the amplitudes of S1 responses while the remainder (Juottonen et al., 2002; Maihöfner et al., 2003; Vartiainen et al., 2008) did find some evidence of greater S1 response for the affected compared to the unaffected side. fMRI evidence is equally mixed: no differences were observed between CRPS patients and healthy controls in S1 activation strength to a variety of stimulations ranging from light touch to tonic pain in two studies (Forster et al., 2000), including no S1 differences in comparing the hyperalgesic (affected) vs. unaffected limbs (Maihöfner et al., 2005). However, CRPS patients with allodynia did have augmented S1 and S2 cortical responses in one fMRI study (Maihöfner et al., 2006). Following this work, Pleger et al. (2006) found overall lower responses in S1 and S2 cortex to tactile stimulation. Hence, the evidence for changes in the amplitude of somatosensory processing in patients with CRPS is inconsistent and so far has not been shown to relate to the degree of cortical reorganization.

If cortical disinhibition and reorganization does not result from changes in afferent inputs in CRPS, then a loss of inhibitory cortical interneurons may occur by another mechanism. A hypothesis gaining traction is neuroinflammation. Evidence supporting the case for neuroinflammatory mechanisms in CRPS includes findings that, first, CRPS patients have elevated levels of the pro-inflammatory cytokines IL-1β and IL-6 in their cerebrospinal fluid, as well as reduced levels of the anti-inflammatory cytokines IL-4 and IL-10 (Alexander et al., 2005). Second, there is evidence for the spread of microglial and astroglial activation within the spinal cord of CRPS patients (Van Rijn et al., 2011), which may exacerbate neuroinflammation. Third, recent evidence suggests that a number of CRPS patients have serum antibodies that interact with autonomic receptors, in particular the alpha-1a adrenergic receptor (Dubuis et al., 2014), beta-2 adrenergic receptor (Kohr et al., 2011), or the muscarinic acetylcholine receptor (Kohr et al., 2011; Dubuis et al., 2014). Serious neuroinflammatory consequences would be expected to arise when autoantibodies against these receptors exudate from blood vessels, together with complement proteins and leukocytes (Cooper and Clark, 2013).

The cause of such neuroinflammation is unclear but could arise from inflammation within peripheral nerves. From both animal and human studies (e.g., Banati et al., 2001), evidence is accumulating that neuroinflammation can spread, either anterograde or retrograde, via axonal projections in the CNS, thereby establishing neuroinflammatory tracks and secondary neuroinflammatory foci within the neuraxis (Cooper and Clark, 2013). Neuroinflammation spreading to second-order synapses in supraspinal centers provides a potent mechanism to destabilize feedback circuits, such as those involved in proprioception, nociception, and autonomic functions, as occurs in CRPS (Cooper and Clark, 2013). A preclinical model of chronic neuropathic pain has implicated glial activation in hyperalgesia (LeBlanc et al., 2011): minocycline injected into the somatosensory thalamus (posterolateral nucleus) reversed both microglial activity and hyperalgesia. However, whether neuroinflammation affects the somatosensory cortex and related functions in patients with CRPS remains unknown. Future studies could assess the extent of cortical reorganization and perceptual disturbance in CRPS in relation to the presence of neuroinflammatory markers and specifically those in somatosensory and motor cortex.

## Neurocognitive Models of Somatosensory Perception

While most studies on the functioning of the cerebral cortex in CRPS have largely focused on early somatosensory processing, it is known that intact somatosensory awareness depends also on the late cognitive stages of neuronal processing (Auksztulewicz and Blankenburg, 2013; Adhikari et al., 2014) and that neurological disturbances of the body scheme can be caused by the frontal abnormalities (Weijers et al., 2013). If so, perceptual disturbances in some patients with CRPS may in fact point to disturbed cognitive-executive functioning among individuals with CRPS. The mechanics of somatosensory perception has begun to be investigated in terms of its dependency on the executive functions of frontoparietal networks as well as the “salience network” including anterior insula and midcingulate cortex. Much of this investigation has been based on “Hierarchical Predictive Coding (HPC)” accounts of perception [for example (Rao and Ballard, 1999; Friston, 2005, 2008)] that may shed light on body misperceptions and neuroplasticity in CRPS. Here, we outline this theoretic approach, and in subsequent sections, we review supporting empirical data from neuroimaging studies of somatosensory perception and finally explore how this perspective could form the basis for identifying neurocognitive phenotypes of patients with CRPS.

Hierarchical predictive coding accounts of perception originate from the work of Hermann von Helmholtz (1962) who proposed that the brain does not represent sensations *per se*, but rather models the causes of those sensations. Because these causes cannot be perceived directly, they must be inferred from sensory data. However, as Friston discussed (Friston, 2003), the problem is that sensations can potentially have multiple causes that interact. Taking an example from vision, the retinal image size can be affected both by object size and distance from the observer. There is therefore inherent uncertainty in the causes of sensory impressions, which the brain must deal with to generate perceptions and guide actions.

One solution to this problem is for the brain’s model of the environment to contain prior expectations about how causes interact, for example the expectation that regardless of the distance from the observer, objects maintain a constant size. As lucidly described by Clark (2013), HPC models depict that top-down expectancy-related information is used to predict and “explain away” the sensory inputs, leaving residual “prediction errors.” These prediction errors then propagate information forward within the system – they report the “surprise” induced by a mismatch between sensory signals and predictions of those signals and serve to update the brain’s virtual model of the causes of those sensations so as to improve the reliability of predictions. Such errors can occur at multiple levels of a processing hierarchy, such that higher-level systems generate predictions about the inputs to lower-level systems on the basis of their role in modeling the causal structure of the world.

This scheme is attractive due to being computationally efficient (i.e., it reflects computations that neurons could feasibly produce) and providing a structure reminiscent of cortical circuits. On the basis of empirical evidence, asymmetrical (forward and backward) connections are thought to relate to specific computational variables within HCP models (e.g., predictions, prediction errors, and “precision” – a concept we come to later). For example, the dynamics of mismatch responses (in which the brain receives sensory inputs that are unexpected in relation to prior inputs) are better described by the minimization of prediction error than by other alternative hypotheses (Garrido et al., 2008; Chennu et al., 2013; Lieder et al., 2013). An important avenue of future research will be to evaluate how well computational models explain dynamic changes in somatosensory perception and neural plasticity and to assess the importance of these models for understanding the pathophysiology of chronic pain.

## The Role of Top-Down Predictions in Chronic Pain

If the HCP framework is correct, optimal perception and behavior depends on minimizing prediction error. This can either be achieved by changing the brain’s predictions to explain sensory input through the act of perception and learning or by actively changing sensory input to fulfill the brain’s predictions by acting on the world. In the latter case, the agent can selectively sample the sensory inputs that it expects. This is known as *active inference*. As Friston explains (Friston, 2010), an intuitive example of this process would be feeling our way in darkness: we anticipate what we might touch next and then try to confirm those expectations.

Selective sampling of sensory data in order to confirm expectations may help to explain why expectations, as formed by prior experiences, have long been known to modify sensory perception, including the perception of pain. However, it is more recently that functional neuroimaging has begun to delineate the mechanisms by which this occurs and to investigate the role of top-down mechanisms in disease states such as chronic pain. For example, pain expectancies trigger anticipatory neural responses (Ploghaus et al., 1999; Brown and Jones, 2008; Brown et al., 2008b; Palermo et al., 2014) that result in changes in perception, emotion, and behavior (Wager et al., 2004; Brown and Jones, 2008; Clark et al., 2008a; Kong et al., 2013; Seidel et al., 2015). Such changes are adaptive for avoiding acute injury but are potentially maladaptive in clinical conditions in which pain is chronic. These observations have inspired a body of work over the last two decades focusing on identifying the neural mechanisms by which cognitive expectancies influence pain perception and exploring these mechanisms in chronic pain populations. For example, somatosensory responses in S1 and S2 are modulated by expectations (Langner et al., 2011). These expectation effects reflect top-down biases, presumably originating from frontoparietal networks that activate during anticipation of stimuli (Brown et al., 2008a; Watson et al., 2009; Kong et al., 2013) and explain multimodal expectancy effects (Langner et al., 2011). Recent evidence suggests, for example, that key nodes of the frontoparietal and salience networks, the dorsolateral PFC and anterior insula cortex (described in more detail below), show aberrant responses during anticipation of pain that are common across chronic pain populations suffering both nociceptive and non-nociceptive (unexplained) pain (Brown et al., 2014). However, to date these approaches have not been applied to understanding top-down mechanisms in CRPS.

Which aspects of HCP are likely to be of relevance to the pathophysiology of CRPS? According to HCP models, ambiguity in sensory input biases perception toward expectations (Dayan and Abbott, 2001). A hypothetical scenario in which expectations may exert a greater-than-normal influence on somatosensory perception is the existence of sensory nerve pathology resulting in greater signal “noise,” i.e., uncertainty in sensory inputs. This could occur in patients with type 1 CRPS for whom there is evidence of small-fiber neuropathy (Van der Laan et al., 1998; Albrecht et al., 2006; Oaklander et al., 2006). Such changes could be potentially monitored with psychophysics and neuroimaging. For example, neurobiological theories inspired by HCP generally ascribe functional asymmetry to ascending and descending connections (Bastos et al., 2012); indeed, a recent study of visual cortex (involving hierarchical processing from V1 to V4) demonstrated ascending prediction errors are related to fast gamma oscillations, while descending predictions are related to slower beta (13–31 Hz) and alpha (8–12 Hz) oscillations (Bastos et al., 2015). On the other hand, there is some evidence for fast gamma-band activity reflecting recurrent connections between the somatosensory and prefrontal cortices during tactile discrimination (Adhikari et al., 2014), although it is unknown whether these findings correspond to the coding of predictions and/or prediction errors. Indeed, higher-level representations and predictions (e.g., those reflecting conceptual or semantic information about expected changes in the environment) are thought to involve lower frequency bands (Correia et al., 2015), consistent with the idea that lower frequencies entrain brain regions across larger spatial and temporal distances in the brain (Canolty and Knight, 2010). Interestingly, greater spectral power in the EEG in the low-frequency delta (< 4 Hz) and theta (4–9 Hz) ranges, localized to both somatosensory and ventral PFC (orbitofrontal cortex), have been found in CRPS patients compared to control subjects (Walton et al., 2010) in a similar region to that showing gray matter atrophy in patients with CRPS (Geha et al., 2008) and that appears to be important for the top-down self-regulation of pain (Woo et al., 2015) – see Figure 1. This points to the intriguing possibility that the somatosensory processing abnormalities in CRPS are mediated by the long-range and low-frequency entrainment across frontal and somatosensory cortices, representing the influence of high-level predictions on somatosensory perception. This view is also supported by fMRI evidence of greater functional connectivity patterns between the post-central gyrus and prefrontal, cingulate and thalamic regions to cold allodynia in pediatric patients with CRPS (Linnman et al., 2013) compared to healthy controls, which persisted after recovery.

## Modeling Recurrent Connections in the Somatosensory System

**Figure 2:**
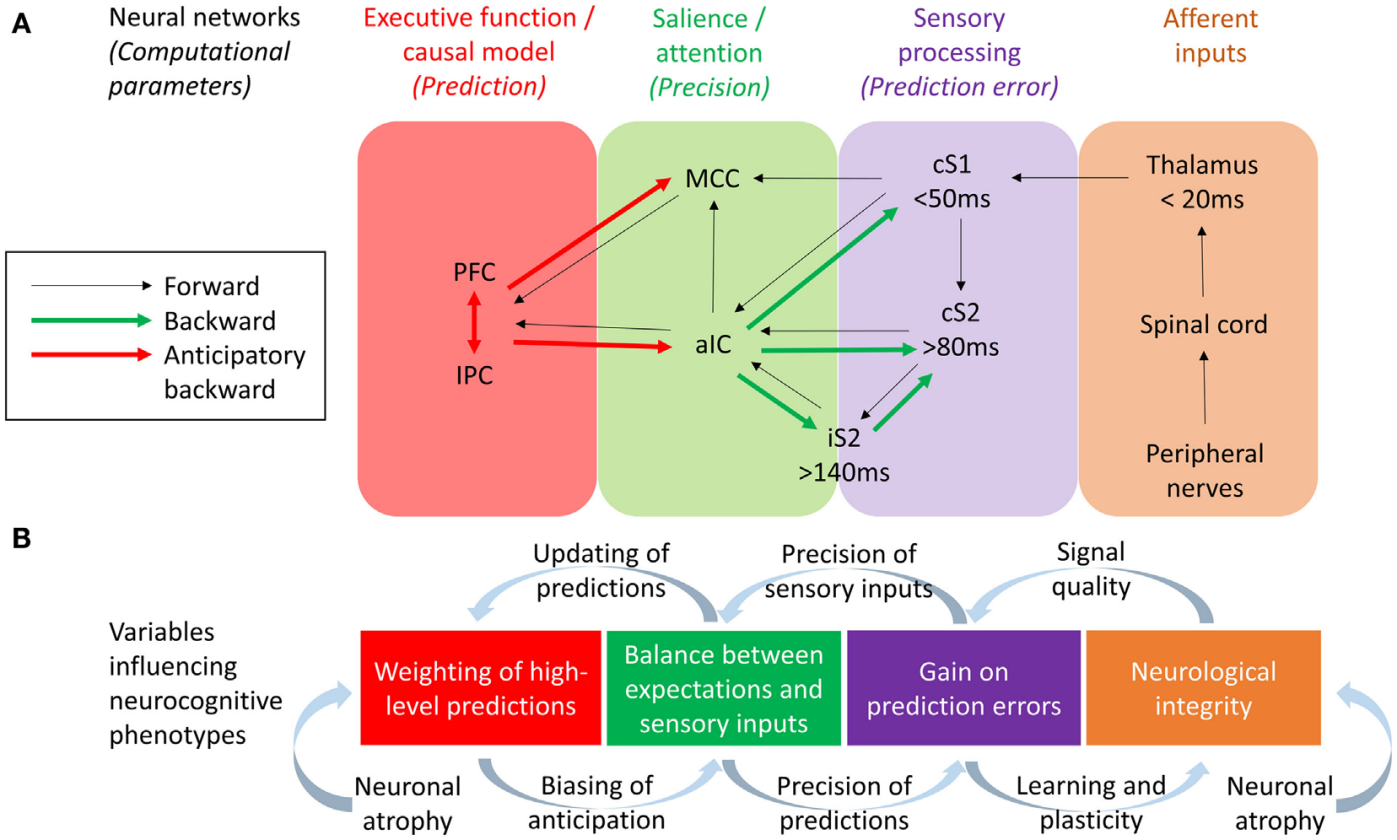
**(A)** Neural networks and their effective connections underlying somatosensory perception, based on Dynamic Causal Modeling research conducted by Allen et al. (2015) and Auksztulewicz et al. (2012) and work studying anticipatory neural activity prior to pain and somatosensation by Brown et al. (2008b), Atlas et al. (2010), and Langner et al. (2011). Frontoparietal executive networks are likely to mediate perceptual predictions while the salience network (aIC and MCC) mediate the effect of predictions on the perception of tactile and pain stimuli, with the aIC acting as a “hub” controlling the balance between bottom-up and top-down information. PFC, refrontal cortex; IPC, Inferior parietal cortex; MCC, Midcingulate cortex; aIC, Anterior insular cortex; iS2, Ipsilateral secondary somatosensory cortex; cS2, Contralateral secondary somatosensory cortex; cS1, Contralateral primary somatosensory cortex. **(B)** Variables hypothesized to influence the neurocognitive phenotype of CRPS, based on a hierarchical predictive coding (HPC) account of parameters describing the computational function of each neural network. The integrity of somatosensory neurons could be potentially influenced both by neurological factors (e.g., neuroinflammation leading to neuronal atrophy) and neurocognitive factors (i.e., changes in neural plasticity related to attention and learning). Resulting changes in signal quality from early cortical processing could change the precision weights attributed to sensory inputs and thereby the gain on prediction errors, a process balanced by the relative precision weights on top-down predictions. According to HCP models, this balance affects the extent to which predictions are updated according to sensory inputs (thereby determining the acuity of tactile perceptions) and also affects the content and influence of top-down predictions as mediated by anticipatory neural activity prior to expected tactile or nociceptive stimuli. Finally, evidence for neuronal atrophy in the executive and salience networks in CRPS lends to the hypothesis of long-term changes in neuroplasticity related to the weighting of top-down predictions, possible leading to aberrant perceptual decision-making.

One approach to investigating the respective roles of somatosensory forward (bottom-up) and backward (top-down) connections in body misperceptions would be through the use of modeling techniques such as Dynamic Causal Modeling (DCM). DCM allows the study the neuronal architecture underlying observed electromagnetic signals (from EEG and MEG) and the effective connectivity between its sources, making it a useful tool in testing alternative models of causal interactions between brain areas that explain the measured data (David et al., 2006). DCM has been applied to EEG data to assess evidence for feedforward, feedback, and recurrent processing between S1 and S2 in a somatosensory detection task (Auksztulewicz et al., 2012) – also see Figure 2. Early ERPs (<80 ms) were well explained by a model assuming only modulation in the feedforward connectivity between S1 and S2 cortices, and this connection was only stronger after stimulus detection for data segments longer than 80 ms. Furthermore, recurrent processing after 80 ms was needed in the model to explain the differences in EEG responses between detected and missed stimuli, and after 140 ms to explain the effect of awareness on ERPs. Therefore, recurrent processing within the somatosensory system, dominated by an enhanced S1–S2 connection, underlies somatosensory detection and awareness. This is consistent with dominant neural models of consciousness suggesting that reportable perceptual experiences depend on (1) sufficient early sensory processing, (2) wide distribution of sensory representations within the executive functions, and (3) recurrent interactions between sensory and frontal brain regions (Lamme, 2006; Dehaene and Changeux, 2011). If so, any reported perceptual abnormality may be caused not only by disturbed sensory processing but also by disturbed executive functions, or abnormal interaction between the sensory and executive regions of the brain. Abnormalities in such recurrent connections may underlie body misperceptions in CRPS.

There is also a body of evidence suggesting an important role for the anterior insula cortex in the anticipation of pain (Porro et al., 2002; Wager et al., 2004; Brown et al., 2008b; Palermo et al., 2014) and mediating the effect of expectations on pain (Koyama et al., 2005; Brown et al., 2008b; Atlas et al., 2010). We have seen that disturbance of body scheme is related to abnormal functioning of insula (Karnath and Baier, 2010), and that this region show atrophy in CRPS patients (Geha et al., 2008 and Figure 1A). An important question is what role the insula plays in neurocognitive (and particularly HCP) models of somatosensory perception and misperception. The insula is a center of salience processing across multiple sensory, emotional, and cognitive domains (Uddin, 2014). The anterior insula is thought to be crucial for the hierarchical processing of bodily information, integrating afferent thalamic and sensory inputs with top-down control signals arising in the prefrontal and cingulate cortex (Seth et al., 2011; Seth, 2013). The right anterior insula is highly interconnected with primary somatosensory areas such as posterior insula and somatosensory cortex (Cerliani et al., 2012; Chang et al., 2013) and anticipates the sensory and affective consequences of pain and touch (Brown et al., 2008b; Lovero et al., 2009). The anterior insula also projects to the amygdala, forming a network contributing to emotional salience (Seeley et al., 2007). Functional connectivity between the insula and amygdala is thought to be related to levels of pain-related fear and is dampened by effective psychological treatment in pediatric patients with CRPS (Simons et al., 2014). Observations of the centrality of the insula in salience processing have led researchers to investigate the role of recurrent connections between the insula and somatosensory cortex in somatosensory perception. DCM has revealed that unexpected somatosensory stimuli increase the strength of forward connections along a caudal to rostral hierarchy – projecting from thalamic and somatosensory regions toward insula, cingulate and prefrontal cortices – reflecting the role of forward connection in conveying prediction error (Allen et al., 2015). The anterior insula, however, was the only region to show increased backwards connectivity to the somatosensory cortex, augmenting a reciprocal exchange of neuronal signals. These results suggest that the anterior insula acts as a hub for regulating somatosensory responses in a top-down manner (Figure 2).

## Neurocognitive Mechanisms of Hemispatial Neglect in CRPS

It has been proposed that the anterior insula and midcingulate cortex form a “salience network” (Seeley et al., 2007). Salience and attention has been linked to the “precision” (reliability/degree of certainty) of sensory inputs (Feldman and Friston, 2010). Within the HCP framework, attention serves the function of balancing top-down and bottom-up influences on perception according to their respective precision weights (Figure 2). In HCP, precision enhances the influence of ascending prediction errors via the regulation of post-synaptic cortical gain (Moran et al., 2013). By this means, attention (via the salience network) can drive learning and appropriate plasticity. By extension of this logic, a lack of precision/attention to a particular limb, i.e., cognitive neglect, may result in a relative loss of cortical function akin to disuse, a hypothetical explanation for cortical changes in patients with CRPS in cases in which no other neuropathology can be observed.

A useful illustration of how this might work in relation to CRPS neglect-like symptoms is the rubber hand illusion (RHI). The RHI refers to the illusory sense of ownership of a plastic hand, which is induced by synchronous tactile stimulation of the fake and the participant’s real (but hidden) hand. In order for the brain to assign the experience of ownership to the artificial hand, certain sensory evidence must be suppressed, namely proprioceptive evidence that the two hands are in different positions (Zeller et al., 2015). In HCP, this corresponds to a reduction in the precision/attention afforded to sensory prediction errors (Feldman and Friston, 2010; Bastos et al., 2012). As evidence in favor of this account, an ERP study (Zeller et al., 2015) identified an attenuation of somatosensory-evoked responses in frontal electrodes that corresponded to cortical sources in the (contralateral) perirolandic area and the parietal lobe. In the absence of an illusion but in the presence of a (perceived) artificial hand, responses were larger in primary somatosensory cortex and inferior parietal lobule. This is consistent with a hypothetical reduction in gain mediated by superficial pyramidal cells in order to resolve the multisensory conflicts arising under the illusion. Should similar multisensory conflicts arise in a patient with CRPS, as implied by the success of mirror therapy in some patients (McCabe et al., 2003), the brain may naturally attempt to resolve these conflicts by attenuating somatosensory predictions errors, with the consequence of driving hemispatial neglect and body misperceptions.

## Neurocognitive Phenotypes

A number of novel neurocognitive mechanisms have been hypothesized here on the basis of the reviewed literature, which if further investigated could help to delineate different phenotypes within the CRPS population. To summarize these possible mechanisms, three hypothetical phenotypes are described here with reference to Figure 2B. This illustrates how different phenotypes could potentially emerge with overlapping symptoms but distinct causes:

1. A patient without sensory misperceptions may experience pain and other overt symptoms for neurological reasons, such as neuroinflammation, which is not severe enough to directly affect neuronal integrity. This patient would be regarded as normal with regard to neurocognitive parameters.
2. A patient experiencing somatosensory misperceptions may have suffered a loss of neuronal integrity, with possible causes including neuroinflammation leading to gray and white matter atrophy in ascending spino-thalamic tracts and/or sensory cortex. This would result in a reduction in signal quality in somatosensory afferents at one or multiple levels from the spinal cord to the thalamus and cortex. A loss of signal quality would result in uncertainty (reduction in precision) regarding sensory inputs and a weighting of perception toward top-down predictions (by increasing the precision of predictions). The result would be a lack of tactile acuity and greater potential for body image distortions arising from abnormally greater biasing of perception by higher-level expectations.
3. A patient may experience somatosensory misperceptions but have no evidence of neuroinflammation or other possible causes of neuronal loss, suggesting the possible influence of other causal factors (e.g., psychological factors). For example, maintenance of abnormally rigid high-level beliefs about the body may require the suppression of somatosensory prediction errors (by decreasing the gain on neuronal error units) in a way akin to the RHI. This would suppress learning and associated neuronal plasticity, with possible consequences for neuronal integrity within sensory cortices.

Both phenotypes 2 and 3 may manifest as signs of body misperception and cognitive neglect, with corresponding cortical changes, but result from a different pathophysiology.

In order to identify these three proposed phenotypes (or others than remain to be hypothesized), three lines of enquiry would need to overlap in future studies, which to date have been investigated separately: the assessment of perceptual distortions, the investigation of neuroinflammation and neuronal loss, and the modeling and estimation of parameters defining causal perceptual mechanisms. Due to the complexity of such investigations and the analytic techniques required to measure these processes (as outlined in this review), in the future, the practicality of identifying such phenotypes in large samples of patients will likely depend on the discovery of practical biomarkers of the different pathophysiological processes.

## Conclusion

There is a large and increasing literature on CRPS for which the present review does not attempt to create exhaustive account. Instead, we have focused on lines of enquiry that we believe are likely to lead toward a more integrated understanding of the pathophysiological mechanisms underlying perceptual disturbances in CRPS. To date, perceptual disturbances in CRPS have largely been investigated separately from neurological deficits, a fact we draw attention to in order to encourage more multi-disciplinary research in this area. Neuroimaging studies have begun to identify potential mechanisms but have lacked an appreciation of the heterogeneity of perceptual disturbances and their potential underlying pathophysiological mechanisms. We suggest that the definition of pathophysiological subgroups of CRPS patients can be achieved by matching specific neurocognitive deficits to cortical mechanisms and demonstrating the effects of specific treatments on those mechanisms.

## Author Contributions

AK conducted and documented literature searches for the first draft. VN wrote sections of the article. NS, SC, and TB critically appraised and edited the article. CB wrote the majority of the article and was responsible for the article’s narrative, structure and editing. All authors approved the final version.

## Conflict of Interest Statement

This review was supported by funding from an EFIC-Grunenthal Grant (awarded to Christopher Brown) and from the Addenbrooke’s Charitable Trust. No potential conflicts of interest have been identified. The other co-authors declare that the research was conducted in the absence of any commercial or financial relationships that could be construed as a potential conflict of interest.

## Abbreviations

aIC, anterior insular cortex; CNS, central nervous system; CRPS, complex regional pain syndrome; DCM, dynamic causal modeling; EEG, electroencephalography; ERP, event-related potential; fMRI, functional magnetic resonance imaging; HCP, hierarchical predictive coding; IL, interleukin; IPC, inferior parietal cortex; M1, primary motor area; MCC, midcingulate cortex; MEG, magnetoencephalography; PET, positron emission tomography; PFC, prefrontal cortex; RHI, rubber hand illusion; S1, contralateral primary somatosensory cortex; S2, secondary somatosensory cortex; TMS, transcranial magnetic stimulation.
